# Identification of Beta-2 as a Key Cell Adhesion Molecule in PCa Cell Neurotropic Behavior: A Novel *Ex Vivo* and Biophysical Approach

**DOI:** 10.1371/journal.pone.0098408

**Published:** 2014-06-03

**Authors:** Keith H. Jansson, Deborah G. Castillo, Joseph W. Morris, Mary E. Boggs, Kirk J. Czymmek, Elizabeth L. Adams, Lawrence P. Schramm, Robert A. Sikes

**Affiliations:** 1 Laboratory for Cancer Ontogeny and Therapeutics, Department of Biological Sciences, University of Delaware, Newark, Delaware, United States of America; 2 The Center for Translational Cancer Research, University of Delaware, Newark, Delaware, United States of America; 3 Department of Biomedical Engineering, The Johns Hopkins University School of Medicine, Baltimore, Maryland, United States of America; 4 Delaware Biotechnology Institute, University of Delaware, Newark, Delaware, United States of America; Queensland University of Technology, Australia

## Abstract

Prostate cancer (PCa) is believed to metastasize through the blood/lymphatics systems; however, PCa may utilize the extensive innervation of the prostate for glandular egress. The interaction of PCa and its nerve fibers is observed in 80% of PCa and is termed perineural invasion (PNI). PCa cells have been observed traveling through the endoneurium of nerves, although the underlying mechanisms have not been elucidated. Voltage sensitive sodium channels (VSSC) are multimeric transmembrane protein complexes comprised of a pore-forming α subunit and one or two auxiliary beta (β) subunits with inherent cell adhesion molecule (CAM) functions. The beta-2 isoform (gene *SCN2B*) interacts with several neural CAMs, while interacting putatively with other prominent neural CAMs. Furthermore, beta-2 exhibits elevated mRNA and protein levels in highly metastatic and castrate-resistant PCa. When overexpressed in weakly aggressive LNCaP cells (2BECFP), beta-2 alters LNCaP cell morphology and enhances LNCaP cell metastasis associated behavior *in vitro*. We hypothesize that PCa cells use beta-2 as a CAM during PNI and subsequent PCa metastasis. The objective of this study was to determine the effect of beta-2 expression on PCa cell neurotropic metastasis associated behavior. We overexpressed beta-2 as a fusion protein with enhanced cyan fluorescence protein (ECFP) in weakly aggressive LNCaP cells and observed neurotropic effects utilizing our novel *ex vivo* organotypic spinal cord co-culture model, and performed functional assays with neural matrices and atomic force microscopy. With increased beta-2 expression, PCa cells display a trend of enhanced association with nerve axons. On laminin, a neural CAM, overexpression of beta-2 enhances PCa cell migration, invasion, and growth. 2BECFP cells exhibit marked binding affinity to laminin relative to LNECFP controls, and recombinant beta-2 ectodomain elicits more binding events to laminin than BSA control. Functional overexpression of VSSC beta subunits in PCa may mediate PCa metastatic behavior through association with neural matrices.

## Introduction

Prostate Cancer (PCa) is the most highly diagnosed non-cutaneous cancer and accounts for the second most cancer deaths among American men [1]. It is estimated that 1 in 6 men will be diagnosed with PCa in their lifetime. In 2014, nearly 233,000 men will be diagnosed with PCa of which nearly 29,500 will succumb to the disease [1]. Early detection of localized PCa allows for treatment options that have pushed five year survival rates to nearly 100% [2]. Unfortunately, the “silent” nature of PCa allows for PCa to proceed undetected until becoming locally or regionally advanced leading to distal metastatic spread of the disease, whereby the 5-year survival rate drops to below 35% [3], [4]. Through a “vicious cycle” of PCa:bone interactions, skeletal metastases engender pathologic fracture and spinal cord compression [5]. Indeed, a majority of men who die from PCa have skeletal metastases [3], [6]–[9]. The current paradigm of PCa metastasis entails PCa glandular egress via the vertebral system of veins known as Batson's plexus [10], [11]. However, the afferent prostatic blood flow does not account for the specificity of PCa metastatic lesion incidence within the spine as only 5–10% of prostatic blood flow is directed towards the spinal cord [10], [11]. Further, the gravitational and muscular-driven flow/counter-flow of the pelvic lymphatics homes to the legs, not the lumbar and sacral regions of the spinal cord [10], [11]. Consequently, we postulate an alternative perineural route of PCa cell prostatic egress.

Prostatic innervation is essential for proper growth, development, and secretory function [12]. The prostate is highly innervated [13], [14] and these nerves connect to the lumbar, thoracic, and sacral regions of the spine, the same regions that bear the highest incidence of PCa skeletal metastases [5]. Thus, these neural pathways may provide direct metastatic routes for PCa cell glandular egress. The phenomenon in which PCa cells spread via nerve is termed perineural invasion (PNI). PNI is defined loosely as cancer cells intimate with nerve by encompassing 33% of the neural perimeter and/or cancer cells within any 3 layers of the nerve sheath [15]. This phenomenon clinically is implicated to play a role in several cancers [15] including breast [16], head and neck [17], pancreatic [18], colon [19], penile [20] and prostate [21]–[26] amongst others. PNI is observed in over 80% of PCa core biopsy specimens [27] and is associated with multiple adverse pathological factors [19], [28]–[36] including stage, grade, and preoperative PSA. Most clinicians recognize PNI as the leading cause of prostatic egress and the mechanism through which PCa penetrates the prostatic capsule [37]; and, PCa cells have been observed in the endoneurium [38], [39] totally independent of lymphatic or vascular involvement [38]. These observations suggest PCa cells interaction with the endoneurium is reliant on neural-specific PCa cell invasion. Analysis of radical prostatectomy specimens reveals that PCa cells and nerves appear to benefit from being in close proximity [21], [24] and co-culturing DRG with LNCaP and DU145 PCa cells *in vitro* demonstrates a direct affinity between PCa and nerve [25]. Despite the benefits that both PCa cells and nerves experience from this intimate relationship, few adhesion molecules have been studied for their role in PNI [23], [40].

The functional expression of VSSCs has been established in numerous malignancies [41], [42]. VSSCs are multimeric transmembrane protein complexes comprised of a pore-forming alpha subunit (α) that is typically flanked by two dissimilar beta (β) subunits on either side [43]. VSSC beta subunits are structurally unique from other voltage sensitive ion channel betas and are part of an entirely different gene family, the immunoglobulin (Ig) superfamily of cell adhesion molecules (CAMs) [44]. Beta subunits, of which there are four isoforms [45] (beta-1, beta-2, beta-3, beta-4), are type 1 transmembrane glycoproteins with an extracellular N-terminus that contains a prototypic V-set Ig domain [46] with similarity to the L1 family of CAMs [47], [48]. Classically, beta subunits are known to modulate gating kinetics and surface expression of VSSC alpha subunits [47]–[51]; in addition, they also have less well-understood homotypic and heterotypic cell adhesion functions [52]–[57]. Interestingly, some of these interactions are independent of alpha subunits [53], [58] while some are not [59]. We have centered this study on beta-2, which is encoded by the gene, *SCN2B* [60].

Beta-2 mRNA [61], [62] and protein are expressed in PCa cells lines with beta-2 protein production exhibiting a significant increase in highly aggressive cells relative to weakly aggressive cells [62]. When beta-2 is overexpressed in weakly aggressive LNCaP cells, their metastasis-associated behavior *in vitro* is enhanced but their subcutaneous tumor volume is reduced drastically [62]. Using beta-2 overexpressing LNCaP cells (2BECFP), vector control LNCaP cells (LNECFP), and highly aggressive C4-2B4 cells, we examined whether the expression of beta-2 altered the neurotropic behavior of PCa cells using a novel *ex vivo* spinal co-culture system, *in vitro* functional metastatic assays, and atomic force microscopy. Interestingly, the level of beta-2 expression has trends with the association of PCa cells with nerve and significantly alters their binding profile, with an exquisite affinity for the most highly expressed extracellular matrix (ECM) CAM in the PNS, laminin.

## Methods

### Ethics Statement

Animal experiments had the ethical approval of the Johns Hopkins University Animal Care and Use Committee (ACUC).

### Beta-2 Amino Acid Sequence Mapping and Analysis

Protein sequences were identified using the online NCBI protein database (www.ncbi.nlm.nih.gov). Potential interacting proteins to *SCN2B* were identified using NCBI pBlast function for basic local alignment. Once alignments were established, a potential 3D model for *SCN2B* was developed by first identifying a template crystal structure using SWISS-MODEL Workspace [49]. Templates were identified similarly for myelin p0 precursor, myelin p0-like 2, *SCN4B* and JAML1. Resultant templates were used further to build a 3D model of *SCN2B* using SWISS-MODEL Workspace. Model proteins were docked using ZDOCK online software [63] and putative binding domains were identified using the Swiss-Pdb Viewer Deepview [64].

### PCa Cell Lines and Maintenance

LNCaP progression model cells [65]–[68], an *in vitro* isogenic cell line lineage that mimics the stages of clinical PCa through progression and development of castration resistance, were cultured in T-medium (Invitrogen, GIBCO,Grand Island, NY) containing 5% (v/v) fetal bovine serum (FBS) (Hyclone, Logan UT) and 1% (v/v) penicillin (10,000 U/ml) Streptomycin (10,000 µg/ml) (Invitrogen) as described originally by Chang et al [69]. Beta-2 overexpression in LNCaP cells was achieved and maintained as described previously [62]. Cell culture media was exchanged every 3 days and cells were split 1∶8 every seven days. A constant temperature of 37°C and 5% (v/v) CO_2_ concentration were maintained in a 95% humidified tissue culture incubator for all cell lines. A day prior to each experiment, old tissue culture media was replaced with fresh media. All cell lines tested negative for Mycoplasma (MycoAlert Mycoplasma Detection Kit, LT07-418 Lonza, Rockland, ME) and were genetically validated (Identifiler, The Core Fragment Analysis Facility, Johns Hopkins Medical Institute, January 2, 2010 and February 26, 2010).

### Preparation and Culture of *Ex Vivo* Organotypic Spinal Cord Co-cultures

Organotypic spinal cord slices were prepared from neonatal (P1-P4) B6.Cg-Tg (Thy1-YFP)16Jrs/J transgenic mice. These mice express yellow fluorescent protein (YFP) in a subset of CNS and PNS neurons and their axons that includes a majority of α-motoneurons [70]. In addition, these axons have been shown to have a strong affinity for and their outgrowth is led by a population of Schwann cells that migrate and proliferate in culture [71]. Spinal cords were dissected and transferred into dissection medium containing Hank's balanced salt solution (Invitrogen, GIBCO, 24020-117, Grand Island, NY), 4.3 mM NaHCO_3_, 10 mM HEPES, 33 mM glucose, 0.03% BSA, 0.15% MgSO_4_-7H_2_O, 100 U/ml penicillin and 100 µg/ml streptomycin. After removal of the dura and the pia externa, the spinal cord was transversely sliced at 350 µm on a Mcllwain tissue chopper (Stoelting Co, 51350, Wood Dale, Illinois). Lower thoracic and lumbar level slices were then cultured on collagen-coated 3.0 µm pore membranes (Corning Inc, 3492, NY) at a density of 5 slices/insert. Inserts were placed in 6-well tissue culture plates with 1 ml of slice culture medium containing 50% MEM (Invitrogen, GIBCO, 10370,Grand Island, NY), 25% Hank's balanced salt solution (GIBCO, 24020–117, Grand Island, NY), 25% Heat Inactivated Horse Serum (Hyclone, Thermo Scientific, SH30074.03, Logan, UT), 25 mM HEPES (J.T. Baker, Avantor Performance Materials, 4018–04, Phillipsburg, NJ), 200 mM L-glutamine (Invitrogen, GIBCO, 25030–081, Grand Island, NY), 3.5 g/L D-glucose, 100 U/ml penicillin and 100 µg/ml streptomycin. Cultures were kept in a humidified chamber with 5% CO_2_ at 37°C. Medium was exchanged every second or third day.

### PCa-Non-myelinated Organotypic Spinal Cord Co-cultures

Prior to adding the PCa cells, cultures were checked to ensure that each well contained at least two or three spinal slices that showed extended axon outgrowth of at least 1 mm in length. This typically occurred when slices were cultured 4-5 days *in vitro* (DIV). After verification of axon outgrowth, either LNECFP (n = 15), 2BECFP (n = 16), or C4-2B4ECFP (n = 17) cells were added in 2 µL droplets at a density of 2,000 cells/ 2 µL. To prepare cancer cells for plating, they were grown to confluence, cell media was aspirated, and cells were washed with sterile 1X PBS for 2 minutes at room temperature. Cells were then detached with 5 mLs non-enzymatic cell dissection solution (Cellgro, Mediatech Inc Manassas, VA) for 1.5-2 minutes at 37°C. Reaction was stopped by addition of 5 mLs of cell media. Cells were then centrifuged for 5 min at 500g at room temperature, supernatant was removed and cells were resuspended in 2 mL of media. Cells were counted and volume adjusted to produce a cell concentration of 2,000 cells per 2 µl of cell media. Notice was taken to place the cells at least 500 µm from the end of the axonal outgrowth. Co-cultures were placed in a 95% humidified chamber with 5% CO_2_ at 37°C. Medium for co-culture was the same as the medium used for spinal slices alone, and medium was exchanged every third day. Co-cultures were incubated for 2 or 6 hours before fixation and processing for immunohistochemistry.

### PCa-Myelinated Organotypic Spinal Cord Co-cultures

For spinal slice cultures used in myelination experiments, slices were first cultured for 7 DIV in slice culture medium, followed by incubation in culture media containing 50 µg/mL ascorbic acid. Incubation with media containing ascorbic acid was maintained for an additional 14–21 DIV to allow for myelination to take place [72]–[74] in these slice cultures. After 28–30 DIV, LNECFP (n = 6), 2BECFP (n = 6), or C4-2B4ECFP (n = 5) cells were added in 2 µL droplets at a density of 2,000 cells/ 2 µL. Procedure for preparation of cancer cells and cell placement was the same as for non-myelinated co-cultures. Co-cultures were incubated for 2 or 6 hours before fixation and immunohistochemistry processing.

### Imaging and Quantification of *Ex Vivo* Organotypic Spinal Cord Co-culture

Co-cultures of organotypic spinal slices with PCa cells were fixed in 4%(w/v) paraformaldehyde (Sigma-Aldrich, P6148, St. Louis, MO) (pH = 7.33) for 2 hours with slow agitation at room temperature. Tissue was then rinsed with 1X PBS and stored at 4°C. Through the eyepiece, each individual spinal slice was examined for axon association with PCa cells. Localization was defined as ECFP engineered PCa cells observed to be in contact with Thy-1 EYFP spinal cord axons. If PCa cells were colocalized with any of the nerve axons protruding from a spinal slice, the slice was noted as being “associated” with PCa cells. If no PCa cancer cells were colocalized with any axons of a spinal slice, the slice was referred to as “unassociated.” The percent association was calculated as associated spinal slices/total number of slices co-cultured with each cell type. The same quantification method was used for both unmyelinated and myelinated nerve axons. Tile scans (4x4, 10x Plan Apochromat) were collected using a confocal microscope (Zeiss LSM 510 NLO, Carl Zeiss MicroImaging) using 514 nm excitation for EYFP and 488 nm laser excitation with a 475–505 nm band pass emission filter for visualization of ECFP.

### F11 Culture and Co-culture Assays

F11 cells, kindly provided by Dr. Jeffrey Twiss of the Nemours AI duPont Hospital for Children, are a hybrid of embryonic rat DRG neurons and a mouse neuroblastoma cell line N18TG-2 [75] and therefore represent a useful model to analyze PCa cell interaction with the PNS. Cells were passaged and maintained in Dulbecco's Modified Eagle Medium (DMEM) (Invitrogen, GIBCO, 11995–065, Grand Island, NY) and supplemented with 10% (v/v) Fetal Bovine Serum (FBS) and 1% penicillin (10,000 U/ml) Streptomycin (10,000 µg/ml) (Invitrogen). To obtain proper differentiation conditions, 1x10^6^ F11 cells were seeded in a 6-well plate and upon reaching 70–80% confluency were serum-starved in DMEM containing 0.5% (v/v) FBS and 10 µm Forskolin (Sigma-Aldrich, F6886-10MG, Allentown, PA). After 24 hours under differentiation conditions, 200,000 of either LNECFP control or 2BECFP beta-2 overexpressing LNCaP cells were seeded on top of the differentiated F11 cells and allowed to adhere for 2 hours. The media in each well was gently replaced and each well photographed at 5 random fields. To stay consistent, the fluorescent images were photographed at the center and four corners of each well on a inverted phase fluorescent microscope (Nikon Eclipse TE2000-U, Nikon Corporation Instruments Company) via ECFP fluorescent filter, with 100x magnification, and an 8 second exposure time. As a background control, wells containing only F11 cells were imaged and yielded little to no background fluorescence. Following image acquisition, PCa cells were counted in each individual well using the Adobe Photoshop CS4 (Adobe Systems Incorporated, Version 11.0.2, San Jose, CA) counting tool and averaged as “PCa Cells Remaining Per Well.” A total of 3 runs in triplicate were completed (n = 3) and a P<0.05 obtained statistical significance.

### Cell Migration Assays

Laminin type I (laminin 111)-entactin free (BD Biosciences, 354239, Bedford, MA) was diluted to a concentration of 0.5 µg/mL in serum free T-medium (Invitrogen, GIBCO, Grand Island, NY). Approximately 2 mL of the newly resuspended laminin solution was pipetted into a 60 mm dish and allowed to sit for 1 hour at room temperature. Subsequently, PCa cells were seeded at a density of 3.75x10^6^ cells in the newly coated dish to attain hyperconfluency after 24 hours. The media was changed the following day and the hyperconfluent 60 mm dish was scratched with a silicon-coated 1 mL pipette tip. A timed series of phase contrast pictures were taken at days 0, 1, 2, 3 and 4 using a calibrated stage micrometer as size reference. Plates were marked so that pictures were taken at precisely consistent positions along each individual scratch. Migration assays were quantified as described previously [62] and a total of five assays (n = 5) were performed with cells ranging from passage numbers 20–24.

### Cell Invasion Assays

Laminin type I (laminin 111)-entactin free (BD Biosciences, 354239, Bedford, MA) was diluted to a final concentration of 50 µg/mL in 1x Phosphate Buffered Saline (PBS). A total of 50 µL of laminin solution was pipetted into the top well of a modified Boyden chamber (Corning, CoStar, 3422, Lowell, MA) and allowed to sit at room temperature for 1 hour in a sterile tissue culture hood. Following coating, 500 µL and 250 µL of complete T-media was placed into the bottom and top wells of the Boyden chamber respectively. Viable PCa cells, 7.5 X 10^4^, were seeded into the top well of the Boyden chamber. Following the 72 hour incubation in a 37°C, 95% humidified incubator with 95∶5, air/CO_2_ mixture, the top of the laminin-coated membrane was swabbed with a cotton swab to remove matrix and cells attached to the top of the membrane. Cells that remained attached to the bottom side of the membrane were stained with DiffQuik (Dade Behring, B4132-1A, Newark, DE), washed with dH_2_O, and quantified as described previously [62]. Assays were run in triplicate a total of 3 times (n = 3).

### MTT Assays

Tissue culture treated 24-well plates were coated with serum free T-medium containing either 0.5 µg/mL laminin type I (laminin 111)-entactin free (BD Biosciences, 354239, Bedford, MA) for 1 hour at room temperature. Vector control LNECFP and beta-2 overexpressing 2BECFP LNCaP cells were seeded at a density of 10,000 cells in 700 µL complete T-medium. The cells were allowed to adhere to the plate overnight with the assay beginning the following day. A total of 300 µL of media was exchanged with fresh media each day. Upon termination of the experiment, 50 µL (3-(4,5-Dimethylthiazol-2-yl)-2,5-diphenyltetrazolium bromide) (MTT) (Invitrogen, Life Sciences, M-6494, Grand Island, NY) was pipetted into each well and incubated at 37°C for approximately 3 hours. The contents of each well and MTT stop solution (50% N,N,dimethylformamide, 20% SDS, in H_2_O) were mixed 1∶1 (v/v) and incubated with slow agitation for 1 hour at room temperature. Absorbance was measured at 568 nm using a FluoStar Optima plate reader (Offenburg, Germany). Assays were done in triplicate at 1, 3, 5, and 7 days with a total of 5 runs (n = 5).

### Production of Secreted Beta-2 Myc-His Ectodomain

The cDNA sequences encoding the full-length coding sequence and ectodomain of beta-2 were amplified via PCR using an identical forward primer 5′-TCGGATCCTTATG CACAGAGATGCCTGGCTACCTCGC-3′, and two distinct reverse primers for full-length 5′-CCCTTCGAACTTGGCGCCATCATCCGGGTTGCCTTC-3′,and ectodomain 5′-CCCTTCGAAGGCCACCGTGGAGTCCCGCTCA-3′, that encompass the signal peptide for beta-2, ensuring the proper trafficking of full-length and truncated beta-2 in mammalian cells. Aside from the primers, the procedure for cloning beta-2 full-length and beta-2 ectodomain is identical. The restriction sites for the enzymes BamH1 and Csp451 within the targeted expression vector, pcDNA 3.1 myc-His A, (Invitrogen, V800-20, Grand Island, NY) were encoded within both primers to allow for sticky end ligation. PCR amplicons subsequently were TA-cloned into pCR2.1-TOPO (Invitrogen, KNM4500-0110, Carlsbad, CA) via topoisomerase according to manufacturer's instructions. Clones were selected via 100 µg/mL Kanamycin in LB Broth, sequence confirmed, and amplified in TOP10 E. coli (Invitrogen, C404010, Carlsbad, CA). The amplicon-containing pCR2.1 TOPO vector and the target vector, pcDNA 3.1 myc-His A, were digested at 37°C overnight using BamH1 (Promega, R602, Madison, WI) and Csp45I (Promega, R657, Madison, WI). Linearized pcDNA 3.1 myc-His A was phosphatase treated using calf intestinal alkaline phosphatase (CIAP) (Invitrogen, 18009-019, Grand Island, NY), phenol/chloroform extracted, and ethanol precipitated while newly digested beta-2 constructs were gel purified from 0.8% agarose using a PureLink Gel Extraction Kit (Invitrogen, K2100-12) and ethanol precipitated. The beta-2 constructs were ligated into pcDNA 3.1 myc-His A for 3 hours at room temperature, transfected into TOP 10 E. coli, and sequence confirmed post-ligation.

### Purification of Beta-2 Ectodomain

Stable beta-2 ectodomain overexpressing cells were generated by transfecting Chinese Hamster Ovary (CHO) cells using DOTAP liposomal transfection reagent (Roche, 1181117 001,Germany) and selected by culturing in DMEM/F12 media (GIBCO) containing 10% FBS and 800 µg/mL G418 sulfate. Serum free DMEM/F12 (GIBCO) conditioned media was collected after 48 hours, 0.22 µm filtered (Millipore, PR02439, Billerica, Massachusetts), and concentrated with a 10,000 MWCO centrifugal filter (Millipore, UFC901008, Cork, Ireland). Beta-2 myc-His ectodomain was purified from conditioned media using Hispur cobalt resin (Pierce, 89966, Rockford, IL) via batch method. A 2 mL microcentrifuge tube was loaded with 300 µL of cobalt resin and then loaded with two resin bed volumes (approximately 600 µL) of equilibration/wash buffer (50 mM sodium phosphate, 300 mM sodium chloride, 10 mM imidazole, pH 7.4). Using a microcentrifuge, the tube was spun 700 X g for 2 minutes and the wash buffer was removed carefully, as to not disturb the resin bed. A total of 1.5 mL of concentrated CHO (control) or CHO Beta-2 (media containing beta-2 ectodomain) conditioned medium was added on top of the resin bed, the resin/media solution was triturated to mix thoroughly, and subsequently incubated on an end over end rotator for 1 hour at room temperature. Following mixing on the rotator, the tube was centrifuged at 700 X g for 2 minutes and the supernatant was saved for post-purification analysis. To remove excess conditioned medium, the resin bed volume was loaded with 1.5 mL wash buffer, triturated, and centrifuged 700xg for 2 minutes. The washing step was repeated a total of 5 times. To elute beta-2 myc-His recombinant ectodomain, the previously washed resin was mixed with 300 µL (one resin bed volume) elution buffer (50 mM sodium phosphate, 300 mM sodium chloride, 150 mM imidazole, ph 7.4) and centrifuged at 700xg for 2 minutes. To ensure all His-tagged protein was eluted, the elution step was repeated a total of five times. Following the 5 wash steps and 5 elution steps, protein concentrations were determined using the nanodrop ND-1000 spectrophotometer on the amino acid setting. The final elutions contained residual imidazole that was dialyzed out using Slide-A-Lyzer Dialysis Cassettes (Thermo Scientific, Rockford, IL). To ensure presence of protein at the appropriate molecular weight, samples were run on SDS-Page gel and visualized via Coomassie stain. Final concentrations were measured post-dialysis using the nanodrop ND-1000 spectrophotometer on the amino acid setting.

### Atomic Force Microscopy

Experiments were conducted using a Bioscope III (Vecco, Santa Barbara, Calif) utilizing silicon nitride DNP-10 Wafer tips (Veeco, A020/09, Plainview, NY) with a 0.06 N/m spring constant. In a chemical fume hood, the tips were treated with Piranha solution (2∶1 H_2_SO_4_:H_2_O_2_) for approximately 5 seconds. Each tip was subsequently washed in dH_2_O and allowed to dry on filter paper in an oven for 10 minutes. After drying, the tips were silanized (95% EtOH (v/v), 5% H_2_O (v/v), 3-Aminopropyl-triethoxysilane, 99% ((Acros Organics, 151081000, New Jersey) (v/v)) for 1 hour at room temperature, rinsed with 100% EtOH, and dried on filter paper in an oven for 5 minutes. Once dried, tips were incubated in 10% glutaraldehyde (v/v) (Electron Microscopy Studies, 16216, Hatfield, PA) for 10 minutes. Following two brief dH_2_O washes, tips for protein:cell studies were coated with 0.01 µg/mL of either perlecan domain IV, laminin type I (laminin 111), or fibronectin as described previously [76]. Young's modulus (tensile modulus) measurements were performed in Force/Volume mode at a rate of 1 Hz with a forward/reverse velocity of 3 µm/sec. A total 2700 engagements over 3 cells (900 engagements per cell) were executed for both LNECFP and 2BECFP cell types. Tips for protein:protein studies were placed in a protein solution containing either 100 µg/mL purified beta-2 ectodomain, 100 µg/mL BSA, 1x PBS with 20% CHO conditioned media (v/v), or 1x PBS with 20% CHO Beta-2 conditioned medium (v/v). Once incubated with appropriate protein, tips for both the protein:cell and protein:protein studies were incubated overnight at 4°C in a humidified chamber. Prior to each experiment, functionalized tips were rinsed in 1X PBS to remove aggregate protein and thermal tune calibrated for both the spring constant (N/m) and deflection sensitivity (nm/V). For whole cell AFM experiments, cells were seeded at 50% confluency in a 60 mm dish 24 hours in advance. The cantilever was slowly engaged to either LNECFP (n = 3) or 2BECFP cells (n = 3), with a total of 3 sites measured per cell and a 5 second surface delay for each engagement. Each cell site was engaged by the tip exactly 300 times, yielding a total of 900 engagements per cell and 2700 total tip:cell engagements per cell type (n = 2700). For protein:protein AFM studies, the tip engaged 600 times to 3 different spots on a 60 mm dish coated with 50 µg/mL laminin type I (laminin 111) with no surface delay for approximately 1800 engagements per tip coating (n = 3). The binding force was measured and generation of force curves was achieved at a frequency of 1 Hz in relative trigger mode. To ascertain patterns in binding force the following parameters were analyzed: force distribution, total binding events, and average binding events per engagement.

### Statistical Analysis

Data are represented as mean ± standard error of the mean (SEM) or fold relative to control ± the square root of the sum of the squares of the normalized standard error. Statistical analysis was performed using a two-tailed Student's t-test. A P<0.05 was considered statistically significant and graphic visualizations exhibiting statistical significance relative to control are denoted via asterisks.

## Results

### Beta-2 Putative Binding Motifs

The putative extracellular binding motifs within beta-2, like all other VSSC betas, lie within its V-set Ig domain. This domain contains residues with highly conserved sequence homology associated with binding (Table 1, bold letters) to the CAMs: Myelin Protein P0 Precursor (MP0), Myelin Protein Zero Like 2 Precursor (MPZL2), *SCN4B* isoform1 precursor (*SCN4B*), JAM L1 isoform 1 precursor (JAML1). The conserved residues between proteins imply a 1/20 chance of interaction. JAML1 is expressed almost exclusively in the hematopoietic system. MPZL2 is highly expressed in the thymus, although it is also expressed in the PNS along with MP0, which is a major component of peripheral myelin. *SCN4B*, the beta-4 VSSC auxiliary subunit, is expressed highly in dorsal root ganglion of the PNS. Fascinatingly, three of the proteins with major sequence conservation to beta-2's putative binding domains are peripheral nervous system proteins.

**Table 1:** BLASTP and Alignment of the amino acid sequence of beta-2 (*SCN2B*) show several proteins having highly conserved domains within their sequence.

Accession	Protein	Putative Interacting Domain
NP_000521.2	Myelin protein P0 precursor	QFRGRVEWVGDISK––HDGSIVIRNLDYIDNG
NP_005788.1	Myelin protein zero-like protein 2 precursor	RFKDRVSWDGNPER––YDASILLWKLQFDDNG
AAQ89304.1	SCN2b	RFQDRVEFSGNPSK––YDVSVMLRNVQPEDEG
NP_777594.1	SCN4B isoform 1 precursor	KDDDRITLVGSTKEKMNNISIVLRDLEFSDTG
NP_001091996.1	JAM isoform 1 precursor	HFQNRVNLVGDIFR––NDGSIMLQGVRESDGG

This may be the region responsible for protein:protein interaction between V-set Ig domain containing proteins. Proteins with high similarity included those within the hematopoietic, immune, and peripheral nervous systems. Letters in bold signify conserved amino acids within the putative interacting domain.

### PCa Cells Associate With Spinal Cord Nerve Axons

**Figure 1:**
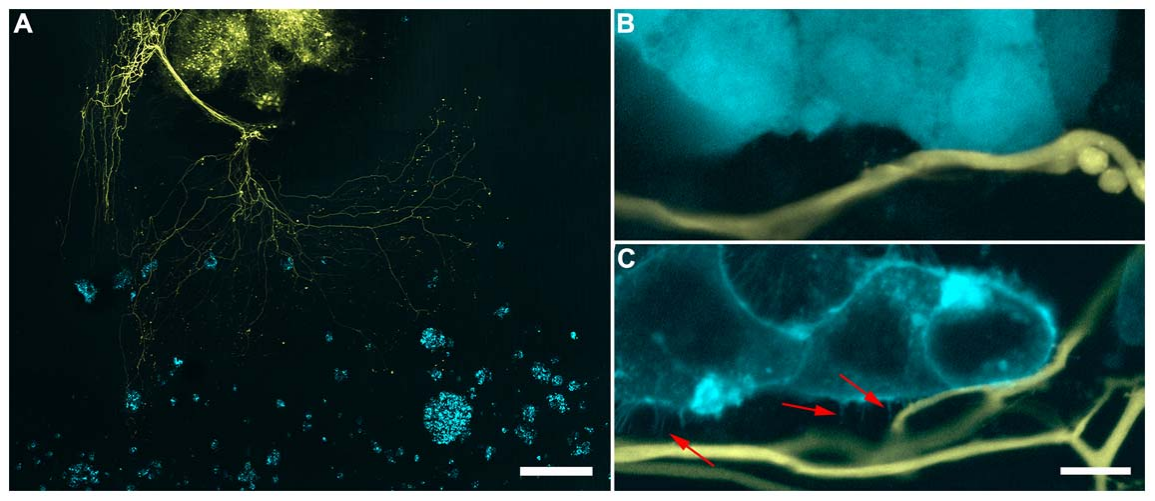
LNCaP cells that overexpress beta-2 generate microprocesses towards nerve while vector control LNCaPs do not. **A**. A tile scan of 2BECFP cells (blue) in co-culture with a YFP+ spinal slice (yellow) displays prostate cancer cells in close proximity to YFP+ nerve axons (scale bar  =  500 µm). **B**. LNCaP vector control LNECFP cells can be closely positioned to spinal cord axons (scale bar  =  10 µm). **C**. 2BECFP cells exhibit what appears to be a membrane-specific CFP fluorescence and most notably, beta-2 specific microvilli (red arrows, scale bar  =  10 µm).

**Figure 2:**
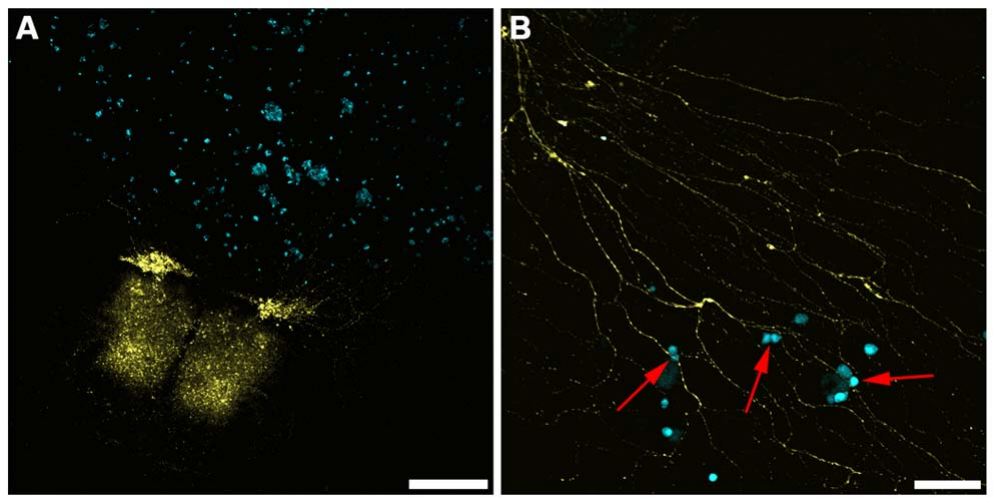
Representative confocal images from novel *ex vivo* organotypic spinal cord slice and co-culture model with C4-2B4 cells. **A**. Tile scan where YFP spinal cord is clearly positive as well as outgrowth of neurons with multiple nodal appearance (scale bar  =  500 µm) **B**. Higher magnification image of neuronal outgrowths from **A**. In this image C4-2B cells appear to associate with YFP+ nerve axons (red arrows, scale bar  =  100 µm).

To examine whether PCa cells have an affinity for spinal cord nerve axons, a novel *ex vivo* organotypic spinal cord co-culture model was developed. Within this model, PCa cells of varying aggressiveness and levels of beta-2 expression were examined for their association with nerve axons. A representative tile scan displays 2BECFP cells in association with YFP+ nerve axons (Figure 1A). At a higher magnification LNECFP cells exhibit close proximity with a YFP+ nerve axon (Figure 1B). 2BECFP cells (Figure 1C) appear to have similar proximity to the YFP+ nerve axon as LNECFP cells (Figure 1B); however, they generate beta-2 specific microvilli that are unique to the 2BECFP cell type (Figure 1C, red arrows). Indeed, LNECFP cells do not produce visible microvilli. C4-2B4 cells transfected with the ECFP vector were observed in propinquity with nerve axons, as exhibited by tile scan (Figure 2A) and high magnification representative images (Figure 2B, red arrows).

### Beta-2 Protein Levels May Enhance PCa Cell Association with YFP+ Spinal Cord Nerve Axons and Nerve Cells

**Figure 3:**
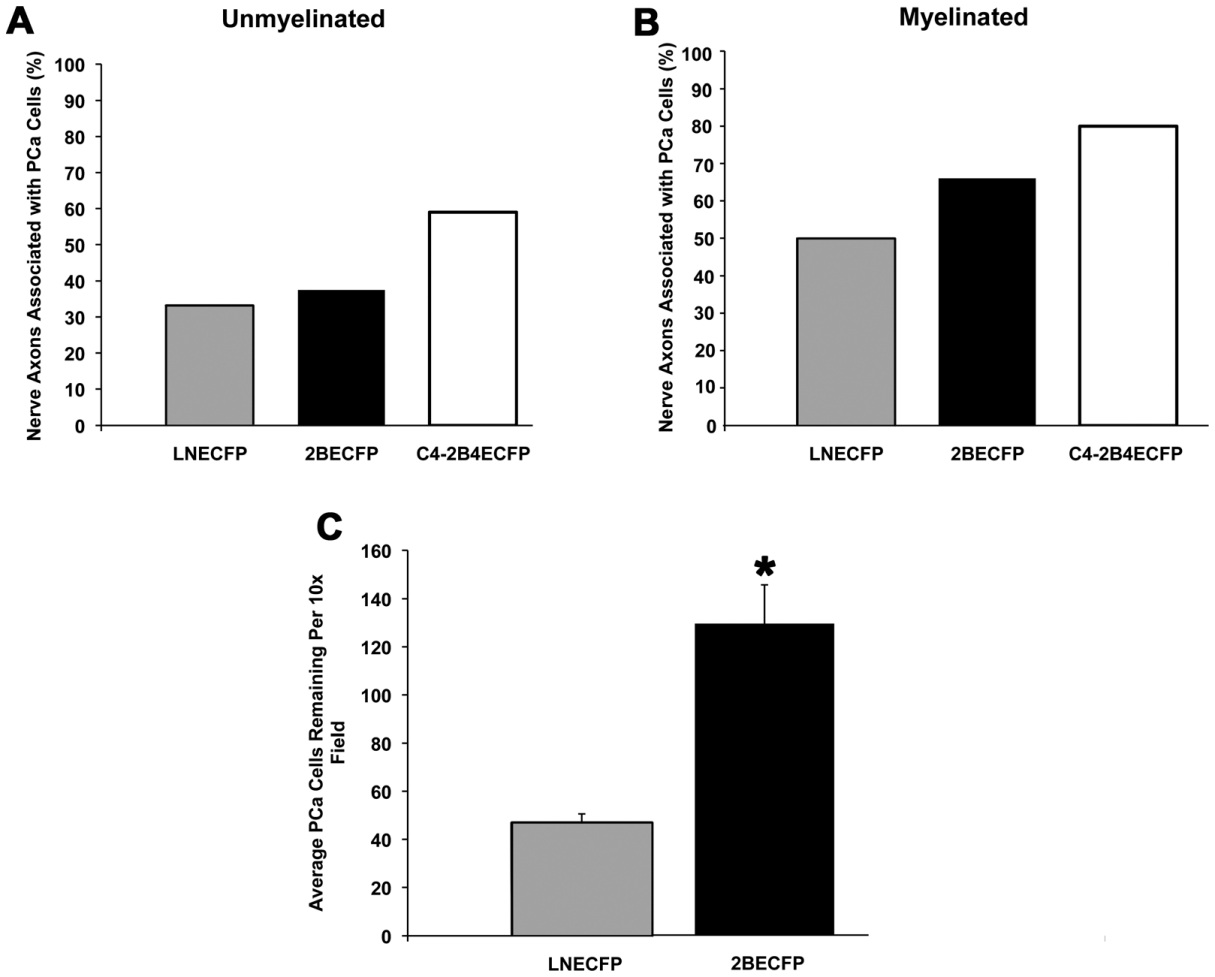
Beta-2 expression increases PCa cell association with nerve. **A**. The association of PCa cells with unmyelinated nerve axons is associated directly with metastatic potential of the PCa cells and increased beta-2 expression. **B**. PCa cell association with nerve displays a similar trend when axons are myelinated. **C**. Following 2 hours in co-culture with neuronally differentiated F11 cells, 2BECFP cells have established more stable associations as compared to LNECFP cells (Per 10x field, 6,156.27 µm^2^). Graph represents the mean ± SEM and the asterisks denote statistical significance (P< 0.05, n = 3).

Regardless of their aggressiveness and/or beta-2 expression, all PCa cells tested in this system associated and/or localized with YFP+ nerve axons (Figure 1 and 2). However, quantitative analysis of tile scans reveals beta-2 levels may affect the ability of PCa cells to associate with YFP+ nerve axons after 2 hours in co-culture. A relative increase in association with YFP+ nerve axons was observed as cells became more aggressive and metastatic and/or expressed more beta-2 (Figure 3A, black bars). To amplify the presence of myelin protein on YFP+ nerve axons, a putative binding partner of beta-2, the cultures were placed under myelinating conditions. Under these conditions and following 6 hours in co-culture, a trend of increased association with enhanced metastatic potential and expression levels of beta-2 was observed (Figure 3B). Utilizing a reductionist approach towards PCa cell association with nerve cells, we examined LNECFP and 2BECFP association with differentiated F11 cells after 2 hours of co-culture. Beta-2 overexpressing 2BECFP cells have a significantly enhanced ability (129.3 cells per 10x field) to remain in co-culture with F11 cells following 2 hours co-culture and subsequent media exchange relative to LNECFP cells (43.3 cells per 10x field) (Figure 3C, P<0.05). As PCa cells become more aggressive and metastatic and express more beta-2, they associate more frequently with both unmyelinated and myelinated spinal cord YFP+ nerve axons and have a significantly enhanced ability to remain in co-culture with differentiated F11 cells.

### 2BECFP Cells Exhibit Augmented Migration on Laminin

**Figure 4:**
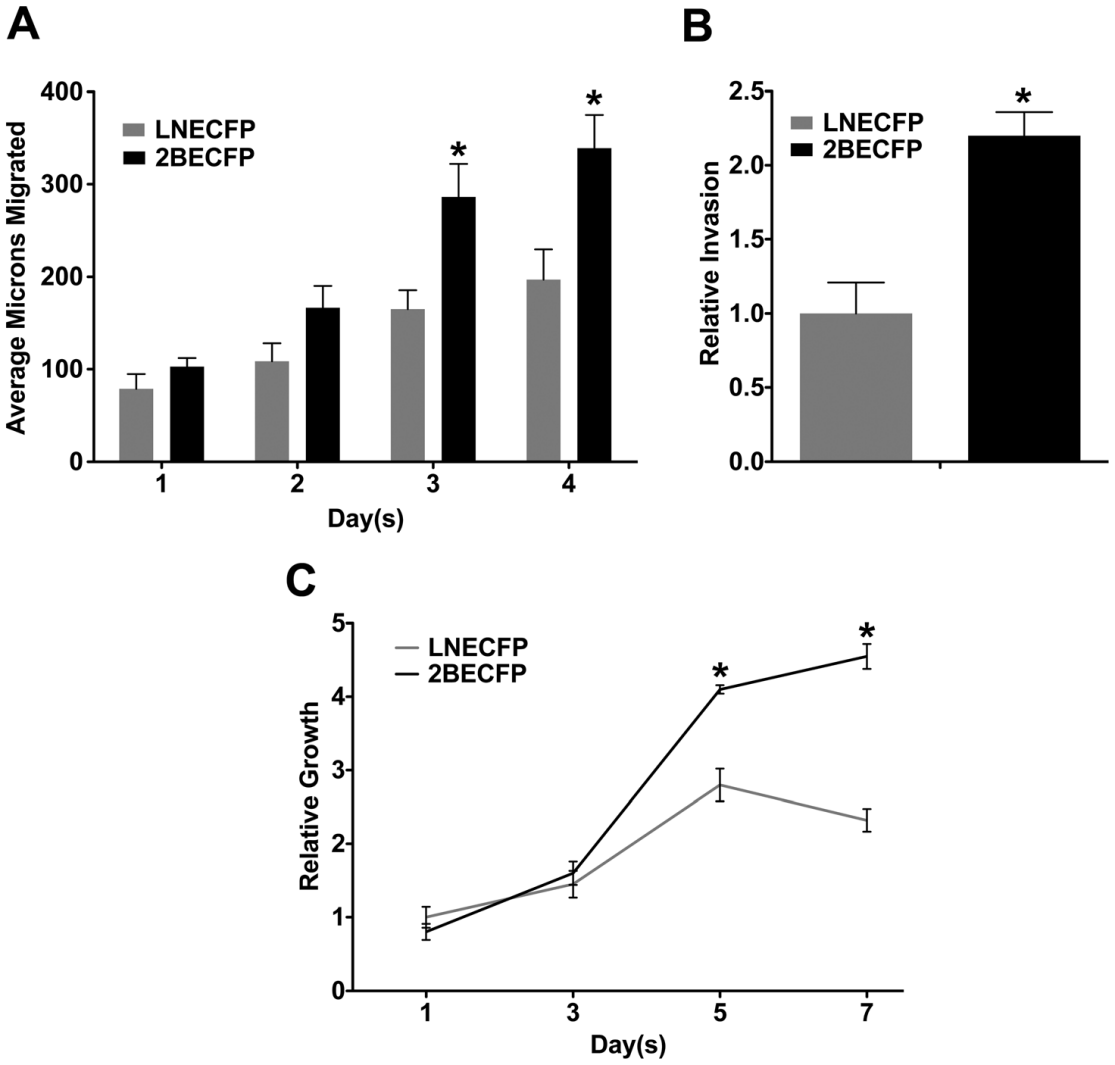
Overexpression of beta-2 enhances LNCaP cell migration, invasion, and growth on laminin. **A**. Quantified results of wound healing migration assays performed on 60 mm dishes pre-coated with 0.5 µg/mL laminin. 2BECFP cells exhibit a trend in increased migration on laminin compared to LNECFP cells beginning at day 1, with statistical significance achieved at days 3 and 4 (P<0.05, n = 5). **B**. LNCaP cells engineered to overexpress beta-2 have a 2.2 fold increase in invasion through 50 µg/ml laminin compared to vector control LNCaPs (P< 0.05, n = 3). **C**. Quantified results of MTT assays on 0.5 µg/mL laminin. 2BECFP cells have a statistically significant growth advantage on laminin relative to LNECFP cells beginning at day 5 (P< 0.05, n = 5).

Aggressive cancer cells have an enhanced ability to migrate and are typically highly motile. As previously demonstrated in scratch assays, beta-2 overexpression enhanced LNCaP cell migration on plastic tissue culture dishes [62]. We examined whether this behavior would be consistent on plates coated with 0.5 µg/mL laminin. A trend in increased migration of 2BECFP relative to LNECFP controls is observed at both 1 day and 2 days with statistical significance achieved at both 3 days and 4 days (Figure 4A, P<0.05).

### Beta-2 Overexpression Enhances LNCaP Invasion Through Laminin

Distal metastasis of cancer cells is facilitated, in part, by the improved ability of cancer cells to invade through basement membranes. As demonstrated previously, 2BECFP cells exhibit increased invasion through Matrigel relative to LNECFP cells over 3 days [62]. Laminin is one of the most abundant extracellular matrix (ECM) molecules in the PNS. Therefore, we determined whether 2BECFP cells displayed augmented invasion through a dense laminin matrix (50 µg/mL). Over a 3-day period, LNCaP cells overexpressing beta-2 had a nearly 2.2 fold increase in invasion relative to the vector control LNECFP cells (Figure 4B, P<0.05). The fold difference between LNECFP and 2BECFP cell invasion through laminin is analogous to the Matrigel invasion experiments conducted previously [62] and may signify beta-2 mediated perineural invasion.

### Overexpression of Beta-2 Confers Late Survival Advantage on Laminin

During PCa progression, PCa cells develop an ability to proliferate within and around the gland. However, when the cells are plated on plastic tissue culture dishes, beta-2 overexpression in LNCaP cells shows no difference from control at days 1, 3, and 5 [62] but only a difference in saturation density. Laminin has been implicated as a molecular binding partner in the facilitation of PCa PNI [40]. To examine whether LNECFP and 2BECFP cells exhibited laminin-specific growth, rates we performed MTT assays on laminin. 2BECFP cells displayed improved growth beginning at day 5 and continuing to day 7 when compared to LNECFP cells (Figure 4C, P<0.05). Thus, overexpression of beta-2 in LNCaP cells augments growth on laminin beginning at 5 days.

### 2BECFP Cells Have a Reduced Young's Modulus Relative to LNECFP Cells

**Figure 5:**
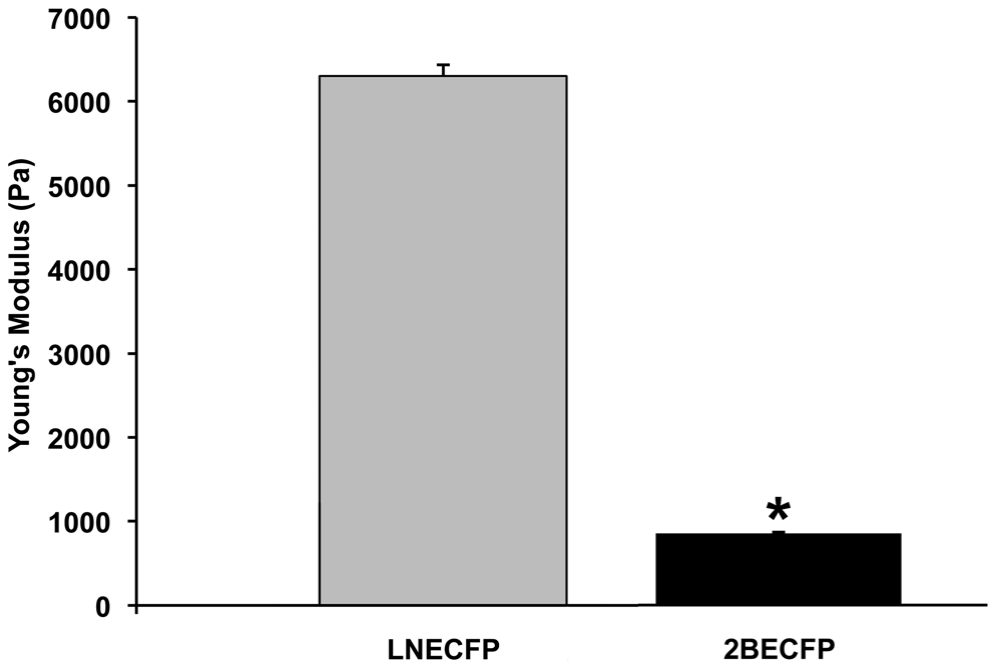
Young's modulus (tensile modulus) of LNECFP and 2BECFP cells. Using an AFM cantilever, the Young's modulus of beta-2 overexpressing 2BECFP cells (850 Pa) was found to be significantly lower than the Young's modulus of LNECFP cells (6294 Pa) (P< 0.05). Data represented as average Young's modulus ± SEM (n = 3).

Recent reports indicate the measurement of a cancer cell's elastic modulus as a technique to determine the metastatic nature and/or aggressiveness of cancer cells via AFM [76]–[81]. Typically, as the aggressiveness of cancer cells increases, their Young's modulus decreases and elasticity increases. 2BECFP LNCaP cells exhibited an average Young's modulus of 850 Pascals (Pa), which is significantly lower than the 6,294 Pa average measurement obtained from their LNECFP counterparts (Figure 5, P<0.05). Thus, overexpression of beta-2 in LNCaP cells results in a reduced Young's modulus and increased cellular elasticity.

### Beta-2 Overexpression Alters LNCaP Cell Binding Force, Total Binding Events, and Binding Force Distribution

**Figure 6:**
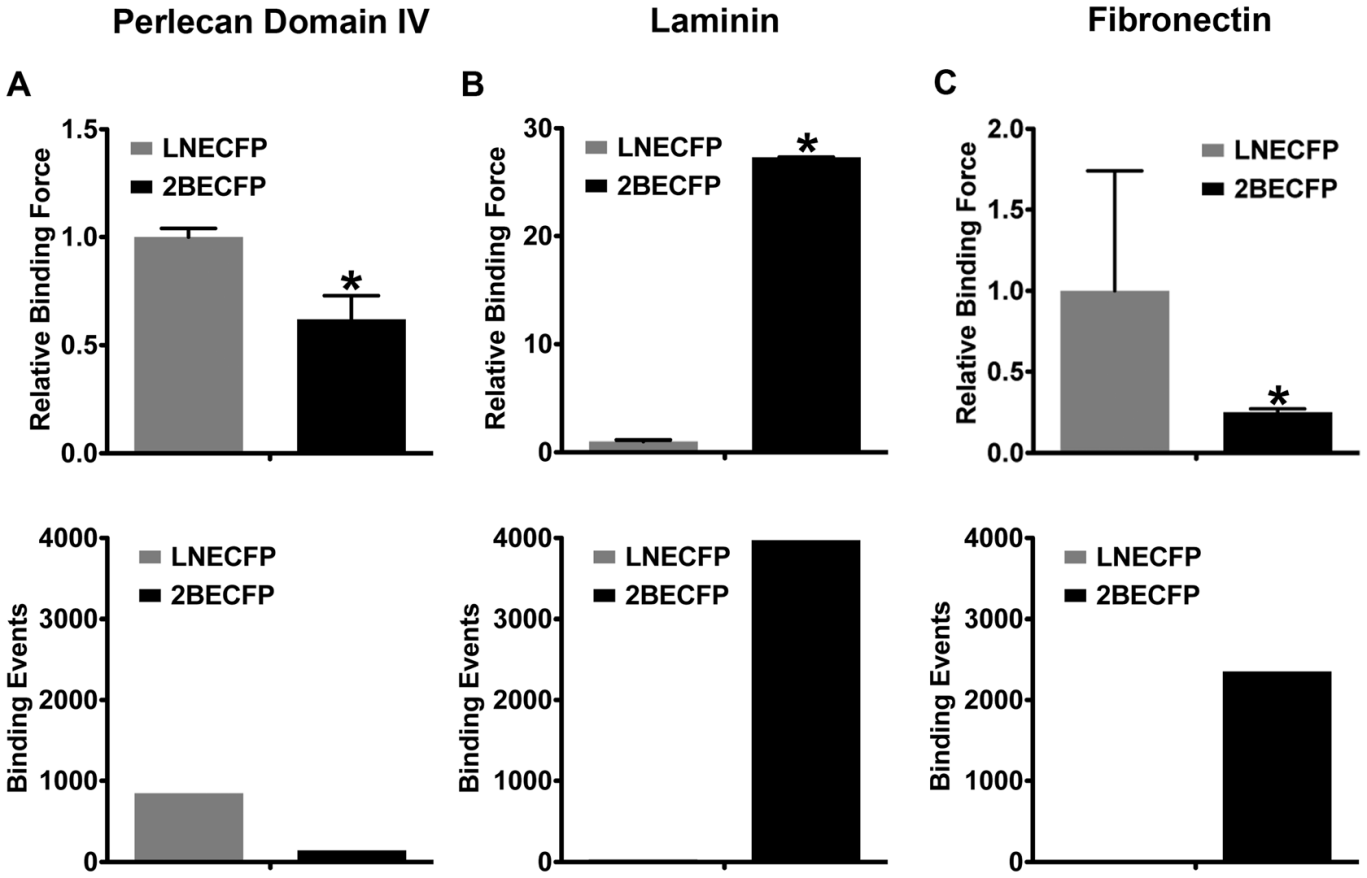
Fold binding force (A, B, C) and total binding events (D, E, F) of perlecan domain IV (A, D), laminin (B, E), and fibronectin (C, F) attached to an AFM tip engaged to either LNECFP or 2BECFP cells plated on a 60 mm tissue culture dish. **A**. When the AFM tip is coated with perlecan domain IV, vector control 2BECFP cells exhibit a 40% decrease in binding force relative to LNECFP cells (top panel, P<0.05), and sustain nearly 700 less binding events than their LNECFP counterparts (bottom panel). **B**. LNCaP cells that overexpress beta-2 have a nearly 26 fold increase in average binding force to laminin over LNECFP cells (top panel, P<0.05) and incur a massive amount of binding events compared to LNECFP cells (bottom panel). **C**. LNECFP cells have an increased average binding force relative to 2BECFP cells (top panel, P<0.05) but exhibit a diminutive number of total events relative to 2BECFP cells when the AFM tip is coated with fibronectin (bottom panel). Fold binding force data presented as fold relative to LNECFP ± normalized SEM (n = 3).

As demonstrated previously, 2BECFP cells have altered adhesion profiles to vitronectin, fibronectin, Matrigel and Matrigel growth factor reduced relative to LNECFP cells [62]. With this in mind, we were curious as to whether 2BECFP cells would also have distinctive binding profiles to various extracellular matrix molecules via atomic force microscopy (AFM) binding assays. LNECFP control cells exhibited a 40% increase in binding force (Figure 6A top panel, P<0.05) and a major uptick in binding events (852 total, 0.47 average) (Figure 6A bottom panel) to perlecan domain IV relative to 2BECFP cells (145 total, 0.08 average). 2BECFP, beta-2 overexpressing LNCaP cells had dramatically increased binding force to laminin (Figure 6B top panel, P<0.05) and a similarly dramatic boost in total binding events (3973 total, 1.47 average) over LNECFP control cells (34 total, 0.037 average) (Figure 6B bottom panel). Fibronectin-coated AFM tips engaged to LNECFP control cells elicited a nearly 70% enhanced binding force (Figure 6C top panel, P<0.05) but exhibited a substantial reduction in binding events (7 total, 0.004 average) (Figure 6C bottom panel) relative to 2BECFP cells (2354 total, 1.30 average). By overexpressing beta-2 in LNCaP cells, binding affinity for different extracellular matrix molecules is remarkably altered.

**Figure 7:**
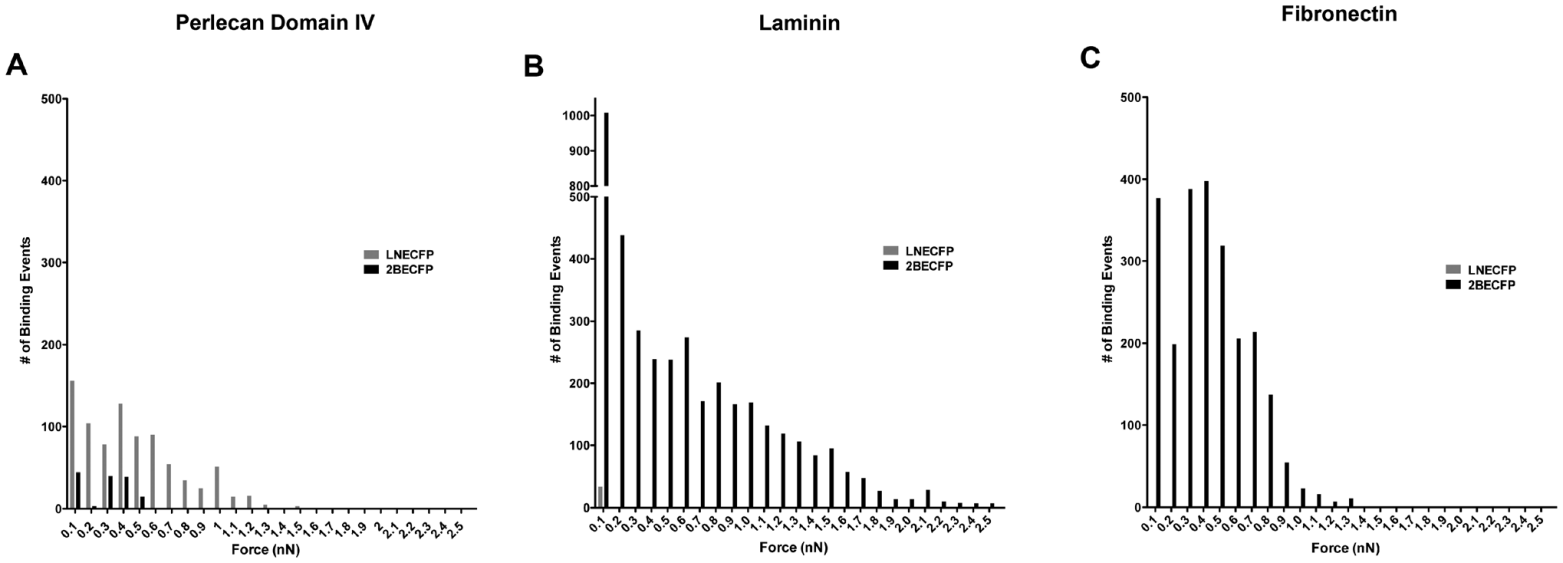
Binding force distribution of perlecan domain IV (A), laminin (B), and fibronectin (C) attached to an AFM tip engaged to either LNECFP or 2BECFP cells plated on a 60 mm tissue culture dish. **A**. Binding between a perlecan domain IV-coated AFM tip and an LNECFP cell displays a more broad binding force distribution than their 2BECFP counterparts. **B**. 2BECFP LNCaP cells display a very wide force distribution relative to LNECFP cells with a laminin-treated AFM tip. **C**. When the tip is coated with fibronectin, LNECFP cells display a narrow force distribution compared to 2BECFP cells (n = 3).

Analysis of binding force distribution allows examination of the frequency of binding events at specific binding forces. We examined whether the binding force distribution of LNECFP and 2BECFP cells was different and whether these differences were matrix-dependent. LNECFP cells displayed a dynamic binding force distribution relative to 2BECFP cells when interacting with perlecan domain IV (Figure 7A), laminin (Figure 7B), and fibronectin (Figure 7C) coated AFM cantilevers. Perlecan domain IV induced a myriad range of binding events with LNECFP cells but a very narrow and shallow binding force distribution for 2BECFP cells (Figure 7A). When coated with laminin, AFM tips educed a large number of binding events through a wide range of binding forces with 2BECFP cells while LNECFP cells incur merely 34 binding events within the 0.1nN range (Figure 7B). Despite the increased average binding force relative to 2BECFP cells measured when LNECFP cells bind to a fibronectin-coated tip, LNECFP binding force distribution was miniscule relative to 2BECFP cells (Figure 7C). Overexpression of beta-2 dramatically alters binding force distribution in LNCaP cells, signifying beta-2 as an influential cog in transient cell adhesion.

### Recombinant Beta-2 Ectodomain Exhibits Binding Affinity to Laminin

**Figure 8:**
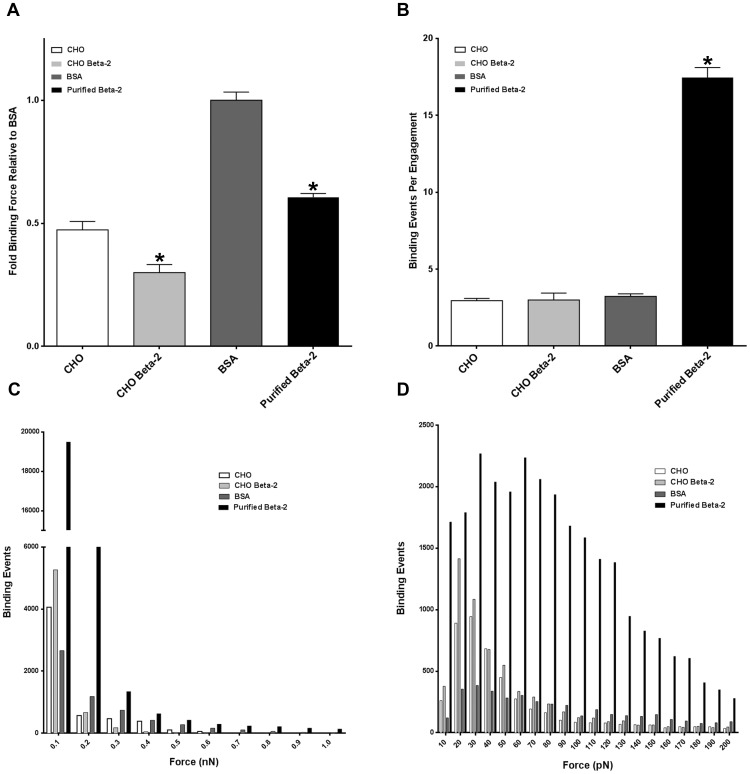
An AFM tip coated with either serum-free CHO cell conditioned media (CHO), serum-free CHO conditioned media containing beta-2 myc-His ectodomain (CHO Beta-2), 100 µg/mL BSA (BSA), or 100 µg/mL purified beta-2 myc-His ectodomain (Purified beta-2) was engaged to a 60 mm plate coated with 50 µg/mL laminin. CHO Beta-2 exhibits a statistically significant reduction in binding force (**A**, P<0.05) relative to control CHO but generates a slight increase average binding events (**B**) and force distribution (**C,D**) when engaged to a laminin-coated dish. Purified beta-2 displays a 40% decrease in binding force relative to BSA (**A**) but elicits a significant increase in average binding events per engagement (**B**) and a more extensive force distribution (**C,D**) than BSA. Specifically, purified beta-2 elicits a significant increase in binding events between 10–200 pN compared to BSA (**D**). Fold binding force data presented as fold relative to BSA ± normalized SEM (n = 3).

Based on the whole cell atomic force studies, it was apparent that beta-2 overexpression induced a strong affinity for laminin by LNCaP cells. To ascertain, at least partly, the cause of the increased binding force and binding events between laminin and 2BECFP cells, we generated a recombinant beta-2 ectodomain. The ectodomain of beta-2 contains the V-set Ig domain including the putative residues for binding to extracellular proteins (Table 1). Atomic force studies utilizing this domain allowed us to determine if beta-2 would bind to laminin directly, or if it was enhancing LNCaP binding to laminin indirectly. Due to the entirely nonspecific nature of BSA interaction with laminin: CHO, CHO Beta-2, and Purified Beta-2 all had a vastly lower fold binding force relative to the control BSA-coated tips (Figure 8A, P<0.05). However, purified beta-2 elicited considerably more binding events (30,856 total, 17.46 average) than CHO (5694 total, 2.95 average), CHO Beta-2 (6229 total, 3 average), or BSA (5804 total, 3.23 average) (Figure 8B). This data is consistent with the observed binding force distribution observed in figure 7, as purified beta-2 had a very large binding force distribution between 0.1nN and 1nN, while the treatments yielded very few interactions after 0.1nN (Figure 8C). This difference is more pronounced when examined between 10 and 200 pN, as purified beta-2 has a radically different binding profile than all other treatments (Figure 8D). Employing purified beta-2 ectodomain AFM binding assays to laminin engenders weak binding forces and a myriad of binding events that is unique from the CHO, CHO Beta-2, and BSA binding profiles. In part, these data may suggest a mechanism for the weak and transient binding force observed at lower amplitudes between 2BECFP cells and laminin.

## Discussion

Beta-2 mRNA and protein levels are associated with enhanced metastatic potential [62]. Furthermore, overexpression of beta-2 in LNCaP cells alters their morphology and aggressive behavior to that of an aggressive cancer cell [62]. Given the recently unveiled role of beta-2 in PCa progression and to better understand the effect(s) of beta-2 expression on PCa cell neurotropic behavior, we developed a novel *ex vivo* organotypic spinal cord:PCa cell co-culture model to study PCa neurotropic behavior. While there is an existing model for studying PCa neurotropic behavior [25], our novel model allows for examination of specific PCa cell interactions with unmyelinated and myelinated nerve axons [72]–[74] and nerve-associated cells [71].

While both LNECFP (Figure 1B) and 2BECFP (Figure 1A and 1C) cells are observed in close proximity to nerve, 2BECFP cells display beta-2 specific microvilli and likely to have an augmented number of PCa cell:nerve contacts compared to LNECFP cells. (Figure 1C, red arrows). Indeed, beta-2 overexpression is associated with increased size and number of microvilli in Xenopus oocytes [60] and augmented process length in PCa cells [62]. Although not statistically significant, beta-2 protein expression levels appear to be coupled to an increased trend in PCa cell association with unmyelinated (Figure 3A) nerve axons. As two myelin proteins were identified to be putative binding partners for beta-2 (Table 1), axons were myelinated to enhance levels of axonal myelin proteins. Indeed, similar to when co-cultured with unmyelinated nerve axons, PCa cells with augmented beta-2 protein expression displayed increased association with myelinated nerve axons (Figure 3B). By developing this model, we may have elucidated a link between beta-2 and PCa cell:nerve axon association. Axon-associated Schwann cells accompany all axonal outgrowth in this *ex vivo* organotypic model [71], and all PCa cells in this study were observed in close proximity to Schwann cells (data not shown). In order to better determine whether beta-2 affects PCa cell interaction with nerve cells, experiments were run with immortalized F11 cells, which closely mimic nerve cell properties i*n vitro* [75]. Following a 2-hour incubation and media exchange, a significantly larger number of 2BECFP cells remained in co-culture relative to LNECFP control cells (Figure 3C). These data suggest that increased levels of beta-2 expression enhance PCa cell affinity to neural cells. These data affirm, in part, observations using the organotypic spinal cord model where increased beta-2 expression trends toward increased PCa cell association with nerve axons (Figure 1 and 2).

We previously demonstrated that the overexpression of beta-2 enhanced LNCaP cell migration and invasion but had no effect on growth after five days [62]. Based on the trend of increased beta-2 expression and enhanced neurotropism observed in our novel co-culture model (Figures 1, 2, 3), we aimed to determine whether beta-2 expression levels affected aggressive behavior of PCa cells when cultured on the predominant PNS CAM, laminin [40]. 2BECFP cells exhibited a statistically significant increase in migration on 0.5 µg/mL laminin beginning at 3 days (Figure 4A, P<0.05) and invaded through 50 µg/mL laminin (Figure 4B,) at a significantly higher rate relative to control. To validate the migration and invasion data, MTT assays were performed on 0.5 µg/mL laminin. Beginning at 5 days 2BECFP cells exhibited a statistically significant increase in growth on laminin (Figure 4C). Prior to 5 days, there was no significant difference in growth, which further validates the increased migration and invasion of 2BECFP cells on/through laminin as these assays run a maximum of 4 and 3 days respectively. In part, these data suggest a role for beta-2 in PCa cell motility on a PNS matrix.

Atomic force microscopy (AFM) is a valuable tool to quantitatively assess the aggressive character of cancer cells [76]–[81]. We found 2BECFP cells were significantly more elastic than LNECFP cells (Figure 5), affirming other reports of decreased cell stiffness with increased cancer cell aggressiveness [76]–[81]. Additionally, coating an AFM tip with protein and engaging the coated tip to a PCa cell allows the direct measurement of binding forces between the PCa cell and the protein. For this study, tips coated with perlecan domain IV, laminin, or fibronectin engendered binding forces. However, tips coated with collagen I and collagen IV did not induce any binding events (data not shown). LNECFP cells appeared to have a higher affinity for perlecan domain IV than their 2BECFP counterparts (Figure 6A). In contrast, 2BECFP cells had dramatically enhanced binding force (26 fold, P<0.05) and binding force distribution when engaged to laminin compared to LNECFP cells (Figure 6B). Fascinatingly, overexpression of beta-2 augments LNCaP cell binding to an abundant PNS protein that is implicated in PCa PNI, namely laminin [40]. Another abundant ECM protein, fibronectin, elicits high average binding forces among LNECFP cells but induces scores of binding events from 2BECFP cells (Figure 6C). Indeed, these data suggest that beta-2 enhances LNCaP cell binding to fibronectin transiently but, whether beta-2 is directly responsible for this binding profile remains to be determined. Through the overexpression of the VSSC CAM, beta-2, the binding force and binding force distribution of LNCaP cells is changed drastically, exhibiting an enhanced tropism for a PNS molecule linked to PNI, namely laminin. Whether Schwann cell associated laminin (laminin 211) will exhibit a differential effect remains to be determined.

To ascertain whether beta-2 is responsible for the increase in 2BECFP binding force and binding force distribution to laminin directly, AFM studies were conducted using the ectodomain of beta-2 (Purified Beta-2) and BSA (BSA) as a non-specific purified protein control. Beta-2 ectodomain was purified from serum-free conditioned media from *SCN2B* ectodomain secreting CHO cells. Therefore, CHO cell conditioned media with (CHO Beta-2) and without (CHO) beta-2 ectodomain also were assayed for binding affinity to laminin. Initial analysis revealed CHO, CHO Beta-2, and Purified Beta-2 to have much lower fold binding force to laminin relative to BSA (Figure 8A, P<0.05). However, BSA binding force to laminin is magnified by the nonspecific profile of BSA binding. Indeed, when the binding force distribution is examined, the affinity of purified beta-2 for laminin is apparent (Figure 8B, 8C, 8D). Purified beta-2 elicits a substantial number of average binding events per engagement compared to CHO, CHO Beta-2, and BSA (Figure 8B). Furthermore, there is a stark difference in the binding force distributions of each tip treatment to laminin. Purified beta-2 exhibits numerous binding forces from 0.1 nN to 1 nN compared to other tip coatings (Figure 8C). This difference is magnified further when observed in the 10 pN to 200 pN range as beta-2 binding to laminin induces a myriad of binding events at a scale to which no other tip treatment ascends (Figure 8D). Although beta-2 is present in transfected CHO cell conditioned medium (Fig S1), the sheer amount of growth factors, proteases, and ECM molecules present in conditioned medium relative to the purified protein fraction may hinder beta-2 binding to laminin. While the binding forces between purified beta-2 and laminin are not large in scale, they are numerous and imply a direct, weak, and transient binding event between beta-2 and laminin in 2BECFP cells that, in aggregate could generate a great deal of specific binding force. Alternatively, the stronger binding force events exhibited by 2BECFP cells may be a result of an obscure indirect mechanism that has yet to be elucidated. Certainly, CAMs containing Ig-loops have been demonstrated to activate integrins [82] and this remains an intriguing possibility. Putatively, beta-2 may activate, increase protein production of, or functionally augment the integrin heterodimer α_6_β_1_, which has a functional association with PCa progression [6], [40], [83].

## Conclusions

We previously demonstrated that beta-2 overexpression in weakly aggressive LNCaP cells conferred morphological changes which resulted in enhanced metastatic behavior *in vitro* and significantly reduced tumor volume *in vivo* [62] that could imply a separation between tumor formation and metastatic behavior. These results are similar to observations of RhoC GTPase in prostate, breast, and pancreatic cancer [76], [84], [85]. In the current study, we elucidated a link between beta-2 expression and PCa cell neurotropic behavior. We pursued this observation and found 2BECFP cells to exhibit hastened rates of migration, invasion, and growth on laminin relative to LNECFP cells. Furthermore, we measured a direct and distinct affinity of 2BECFP cells and purified beta-2 ectodomain for laminin via AFM. Although beta-2 has a clear role in PCa cell neurotropism *in vitro*, whether beta-2 facilitates PCa cell perineural metastasis *in vivo* has yet to be determined. The implication of a beta-2 in neurotropic metastasis associated behavior provides valuable insight for the role of VSSC auxiliary CAMs in a poorly understood and ominous aspect of malignant disease.

## Supporting Information

Figure S1 Western blot analysis displays beta-2 protein in transfected CHO cell conditioned medium and in the subsequent affinity purified protein fraction.
(TIF)
