# Supplementary figures and images for: Identification of Beta-2 as a Key Cell Adhesion Molecule in PCa Cell Neurotropic Behavior: A Novel Ex Vivo and Biophysical Approach
Source: PLoS One. 2014 Jun 3;9(6):e98408. doi: 10.1371/journal.pone.0098408 (PMC4043823; doi:10.1371/journal.pone.0098408)

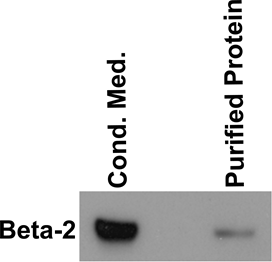

Supplement: Figure S1 — Western blot analysis displays beta-2 protein in transfected CHO cell conditioned medium and in the subsequent affinity purified protein fraction. (TIF) [file pone.0098408.s001.tif]
